# Expression Profiles and Biological Roles of miR-196a in Swine

**DOI:** 10.3390/genes7020005

**Published:** 2016-01-22

**Authors:** Xiaomin Ning, Shuai Liu, Yang Qiu, Guoxi Li, Yanjie Li, Meihang Li, Gongshe Yang

**Affiliations:** 1 Laboratory of Animal Fat Deposition and Muscle Development, College of Animal Science and Technology, Northwest A & F University, Yangling 712100, China; xmning@hotmail.com (X.N.); yingwuzhe01@yeah.net (S.L.); qiu8355643@163.com (Y.Q.); liyanjie1979@163.com (Y.L.); limeihang@163.com (M.L.); 2 College of Animal Science and Veterinary Medicine, Henan Agricultural University, Zhengzhou 450002, China; liguoxi0914@126.com

**Keywords:** miR-196a, swine, expression, adipocyte, proliferation, differentiation

## Abstract

MicroRNAs (miRNAs) are a class of small non-coding RNA molecules, which play important roles in animals by targeting mRNA transcripts for translational repression. Recent studies have demonstrated that miRNAs are involved in regulation of adipocyte development. The expression of miR-196a in different porcine tissues and developing fat tissues was detected, and gene ontology (GO) term enrichment was then used to predict the expression profiles and potential biological roles of miR-196a in swine. To further verify the roles of miR-196a in porcine adipocyte development, a recombinant adenovirus encoding miR-196a gene (Ad-miR-196a) was constructed and used to study the effect of miR-196a on preadipocyte proliferation and differentiation. Here, our data demonstrate that miR-196a displays a tissue-specific expression pattern and has comprehensive biological roles in swine, especially in adipose development. In addition, overexpression of miR-196a had no effect on preadipocyte proliferation, but induced preadipocyte differentiation by increasing expression of adipocyte specific markers, lipid accumulation and triglyceride content. These data represent the first demonstration of miR-196a expression profiles and roles in swine, thereby providing valuable insight into the functions of miR-196a in adipocyte biology.

## 1. Introduction

MicroRNAs (miRNAs) are small non-coding RNA molecules (19–22 nucleotides in length) that regulate gene expression at the post-transcriptional level by targeting mRNA transcripts for cleavage or translational inhibition [1,2]. Biochemical and genetic studies have confirmed that miRNAs are involved in regulation of multiple biological processes, including cell proliferation, differentiation and apoptosis [3], organ development [4], lipid metabolism [5] and tumorigenesis [6]. However, the functions of most miRNAs in swine are still unclear.

Recently, more and more miRNAs were reported to regulate adipocyte differentiation in mouse [7,8,9,10,11] and human [12,13,14]. However, the functions of miRNAs in porcine adipogenesis are seldom reported. To identify the potential miRNAs regulators in porcine adipocyte development and metabolism, solexa sequencing was performed with adipose tissues from piglets and adult pigs. Among the identified miRNAs, a robust increase in miR-196a expression was observed in adipose tissues from adult pigs compared with piglets, which suggests miR-196a may play important roles in adipose tissue development.

Mature miR-196a was first found to play important roles in mammalian limp development and embryogenesis by targeting the homeobox gene clusters [15,16]. In addition, miR-196 was found to be involved in regulation of axolotls tail regeneration [17], immunology, inflammation and virus defense [18,19]. Recently, miR-196a was shown to be differently regulated during tumorigenesis and to be involved in regulating key pathways such as AKT signaling [20], BMP [17] and WNT signaling [21]. Furthermore, overexpression of miR-196a has been reported to inhibit proliferation and stimulate osteogenic differentiation of human adipose tissue-derived mesenchymal stem cells [22]. However, little is known about the expression and functions of miR-196a in swine.

To explore the expression profiles and roles of miR-196a in swine, expression pattern examination, bioinformatics function prediction and gain-of-function analysis of miR-196a were carried out with swine tissues and primary preadipocytes. Our results indicated that miR-196a exhibits developmental and tissue specific expression patterns, and appears to participate in multiple biological processes in swine. Moreover, miR-196a was demonstrated to be involved in regulation of porcine adipose development, and overexpression of miR-196a was shown to promote porcine adipocyte differentiation without affecting proliferation. This research identifies new roles of miRNAs in the regulation of porcine adipose biology.

## 2. Materials and Methods

### 2.1. Reagents

DMEM/F-12 (Dulbecco’s Modified Eagle Medium/Ham’s F-12), Fetal Bovine Serum (FBS), Collagenase Type I were purchased from Gibco (Grand Island, NY, USA). Restriction enzyme PmeI and PacI were bought from New England Biolabs (Ipswich, MA, USA). Lipofectamine 2000 was obtained from Invitrogen (Carlsbad, CA, USA). C/EBPα (CAAT/enhancer binding protein α), PPARγ (peroxisome proliferator-activated receptor γ), FABP4 (fatty-acid-binding protein 4), LPL (lipoprotein lipase) and β-actin antibodies were purchased from Santa Cruz Biotechnology (Santa Cruz, CA, USA). Oil Red O and Methylthiazolyldiphenyl-tetrazolium bromide (MTT) were bought from Sigma-Aldrich Co. (St. Louis, MO, USA). TRIZOL reagent, PrimeScript RT-reagent Kit, and SYBR Premix Ex Taq^TM^ II were obtained from TaKaRa Biotechnology (Takara Biotechnology Co., Ltd., Dalian, China).

### 2.2. Collection of Animal Tissue Samples

Male crossbred piglets (3-days-old) and adult pigs (180-days-old) (*Sus scrofa*, Duroczz × Seghers) were supplied by the experimental farm of Northwest A & F University (Yangling, Shaanxi, China). Ten healthy male crossbred piglets and 10 male crossbred adult pigs were used for tissue samples collection. All experimental animals were treated in accordance with the guidelines of Northwest A & F University Animal Care Committee. The heart, liver, spleen, lung, kidney, skeletal muscle, and subcutaneous adipose tissue were isolated, collected, and quickly frozen in liquid nitrogen, and stored at −70 °C for further experimental application.

### 2.3. Solexa Sequencing and Analysis

For solexa sequencing, adipose tissues from six piglets and six adult pigs were pooled, respectively. Total RNA was then isolated and used for construction of small RNA (sRNA) libraries with Small RNA Sample Prep Kit (Illumina Inc., San Diego, CA, USA). The Solexa sequencing and analysis were then performed with Illumina Genome Analyzer (Illumina, San Diego, CA, USA) as previously described [23].

### 2.4. Target Prediction and Gene Ontology Enrichment Analysis

The TargetScan algorithm [24] and PicTar [25] was used to predict miR-196a targets. *Sus scrofa* genes are not involved in the current version of TargetScan and PicTar, and, therefore, the prediction was based on the mRNA–miRNA interactions of *Homo sapiens*. The predicted targets were then used for Go term enrichment analysis and KEGG pathway analysis with DAVID. Go annotations were downloaded from the Go consortium website [26,27]. 

### 2.5. Cell Culture

Subcutaneous adipose tissue was collected under sterile conditions from the neck and back of the piglets. Primary porcine preadipocytes were obtained as previously described [28,29,30]. Cells were seeded in culture plates at a density of 5 × 10^4^ cells/cm^2^ and maintained at 37 °C in a humidified atmosphere including 5% CO_2_. The culture medium was changed every two days.

### 2.6. Plasmid Construction

The recombinant adenovirus vector encoding miR-196a (Ad-miR-196a) was constructed as previously described [31]. After packaging and amplifying with 293A cells, adenovirus was collected and virus titer was detected by TCID50. The adenovirus was then used to infect porcine primary preadipocytes. An empty adenovirus vector (Ad-Null) was constructed as control. 

### 2.7. Oil Red O Staining and Extraction

Adipogenesis of porcine preadipocytes was evaluated by Oil red O staining and extraction as previously described [32]. Red-stained adipocytes were photographed with microscope and quantified by examining its spectrophotometric absorbance at 500 nm using a UV-2102 PC ultraviolet spectrophotometer (Unico Instrument Co., Ltd., Shanghai, China).

### 2.8. Cell Proliferation Assay

Cell proliferation was assessed with MTT assay. Cells were seeded into 96-well culture plates at a density of 5 × 10^4^/cm^2^ and incubated in 200 μL DMEM/F12 medium containing 10% FBS. At each specific time point, the medium was removed and cells were incubated in 20 μL MTT solution (5 mg/mL) for 4 h. MTT solution was then discarded and 200 μL DMSO was added to dissolve formazan crystal. The solubilized formazan product was spectrophotometrically quantified using DG5031 ELISA Reader (Medical Equipment Co., Ltd., Huadong Electronics Group, Nanjing, China). 

### 2.9. mRNA and miRNA Quantification by qPCR

Total RNA was prepared from frozen tissue or cells using TRIzol reagent (Takara Biotechnology Co., Ltd., Dalian, China) according to the manufacturer’s protocol. Total RNA was quantitated and reverse transcribed with PrimeScript RT-reagent Kit for RT-PCR (Takara Biotechnology Co., Ltd., Dalian, China). Quantitative PCR was performed using the Bio-Rad iQ5 real-time PCR detection system using SYBR Premix Ex TaqTM II (Takara Biotechnology Co., Ltd., Dalian, China). The Bulge-LoopTM miRNA qRT-PCR primer set for miRNAs detection were obtained from Guangzhou RiboBio Co. (Guangzhou, China), and U6 was used as internal control. For adipocyte marker genes, including PPARγ, C/EBPα, FABP4 and LPL, expression was normalized to β-actin. Primers for qPCR were (F forward, R reverse): miR-196a-1 F: 5′-CCGCCTAGGTAGTTTCATGTTGT-3′, R: 5′-AATCCATGAGAGATCCCTACCG-3′. U6 F: 5′-ATTGGAACGATACAGAGAAGATT-3′, R: 5′-GGAACGCTTCACGAATTTG-3′. PPARγ F: 5′-AGGACTACCAAAGTGCCATCAAA-3′, R: 5′-GAGGCTTTATCCCCACAGACAC-3′. C/EBPα F: 5′-TGTTGGGGATTTGAGTCTGTG-3′, R: 5′-GGAAACCTGGCCTGTTGTAAG-3′. FABP4 F: 5′-GAGCACCATAACCTTAGATGGA-3′, R: 5′-AAATTCTGGTAGCCGTGACA-3′. LPL F: 5′-GGAGAGAGGAAGGGAAAACAGAG-3′, R: 5′-AGACCGACCAATAAACTGCAAAG-3′. β-actin F: 5′-GGACTTCGAGCAGGAGATGG-3′ R: 5′-AGGAAGGAGGGCTGGAAGAG-3′.

### 2.10. Western Blotting

Cell extracts were lysed with lysis buffer (0.5% Triton X-100, 2 mM EDTA, 150 mM NaCl, 1 mM PMSF, 50 mM Tris-HCl at PH 7.5). After BCA protein assay (Thermo Scientific, Waltham, MA, USA), proteins were separated by SDS-PAGE gel, and transferred onto PVDF membranes (Millipore Corporation, Bedford, MA, USA) and immunoblotted with antibodies specific for PPARγ, C/EBPα, FABP4, LPL and β-actin (Santa Cruz Biotechnology, Santa Cruz, CA, USA).

### 2.11. Statistical Analysis

All data are presented as mean ± SEM and were determined by Student’s t-test or ANOVA. The differences were indicated as follows: * = *p* < 0.05, ** = *p* < 0.01 and *** = *p* < 0.001.

## 3. Results

### 3.1. Expression of miR-196a in Normal Porcine Tissues

**Figure 1:**
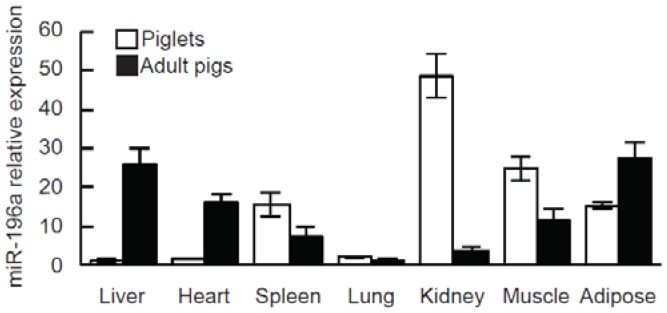
Expression of miR-196a in swine tissues from 3-day-old piglets and 180-day-old adult pigs. Total RNA was isolated from seven different tissues including heart, liver, spleen, lung, kidney, skeletal muscle and subcutaneous adipose tissue, and the expression of miR-196a was analyzed by qPCR and normalized to U6.

In a deep sequencing of the miRNAs involved in the development of swine adipose tissue, miR-196a expression was first found to be significantly upregulated in adult pig adipose tissue compared with the piglets (Table 1), which indicated that miR-196a may play an important role in adipocyte development. To verify the expression of miR-196a, which was identified in the sequencing results, miR-196a expression levels were detected with qPCR in several porcine tissues including adipose, liver, muscle, heart, spleen, lung and kidney. As shown in Figure 1, miR-196a was widely expressed in various tissues from 3-day-old piglets and 180-day-old adult pigs. However, the expression levels of miR-196a varied markedly among different tissue samples. In the piglets, miR-196a was highly expressed in the kidney and muscle, moderately expressed in the adipose and spleen, and lowly expressed in the heart, lung and liver. Whereas, in adult pigs, the highest miR-196a levels were found in the adipose tissue and liver, moderate levels were detected in the heart, spleen and muscle, and low levels were observed in the kidney and lung. Interestingly, contrary to the low expression in piglets, miR-196a was found to be highly expressed in adipose tissue and liver from adult pigs. These results suggest that miR-196a appears to be tissue-specific and to display differential expression patterns in piglets and adult pigs, which suggest miR-196a may have important biological functions in the regulation of pig development. 

**Table 1:** Expression of miR-196a in developing adipose tissues of swine.

miRNA Name	Sequencing Count		Fold Change log2 (Adult Pigs/Piglets)	*p*-Value	Sig-Label
	Piglets Adipose Tissue	Adult Pigs Adipose Tissue			
ssc-miR-196a	125	17,867	7.6132	0	**

Porcine adipose tissues were collected from the backfat of 3-day-old piglets and 180-day-old adult pigs. After RNA isolation, samples were sequenced using Solexa technology. *p*-value: reflects the significance of miRNA differential expression between samples. Sig-label: significance label. *: Fold-change (log2) > 1 or fold-change (log2) < −1, and 0.01 ≤ *p*-value < 0.05. **: Fold-change (log2) > 1 or fold-change (log2) < −1, and *p*-value < 0.01.

### 3.2. Biological Functions of miR-196a Based on Targets Analysis

Most miRNAs are well conserved in evolution, including mir-196 family, which are conserved and localized within the Hox gene clusters among vertebrates [33,34]. In *Sus Scrofa*, four mir-196 precursor sequences have been identified including mir-196a-1, mir-196a-2, mir-196b-1 and ir-196b-2 [15,16,35]. Among the precursors, mir-196a-1 and mir-196a-2 share the same functional mature sequence miR-196a, whereas mir-196b-1 and mir-196b-2 have two mature sequences: miR-196b-3p and miR-196b-5p (Table 2) [36,37] In addition, the same mature sequences of miR-196a from the miRBase database were observed within *Homo sapiens*, *Mus musculus* and *Sus scrofa*, suggesting miR-196a was highly conserved across species and may play similar roles within the species mentioned above. 

**Table 2:** miR-196a genes and mature sequences.

miRNAs	Gene Location	Mature Sequences
ssc-mir-196a-1	Chromosome 12 (24,834,774-24,838,853)	UAGGUAGUUUCAUGUUGUUGGG
ssc-mir-196a-2	Chromosome 5 (19,652,956-19,657,062)	UAGGUAGUUUCAUGUUGUUGGG
ssc-mir-196b-1	Chromosome 18 (49,834,543-49,838,622)	UAGGUAGUUUCCUGUUGUUGGG
		CGACAGCACGACACUGCCUUCA
ssc-mir-196b-2	Chromosome 18 (50,035,507-50,039,584)	UAGGUAGUUUCCUGUUGUUGGG
		CGACAGCACGACACUGCCUUCA

The mature sequences of the ssc-mir-196a-1, ssc--mir-196a-2, ssc-mir-196b-1 and ssc-mir-196b-2 were acquired from the miRBase sequence database.

**Figure 2:**
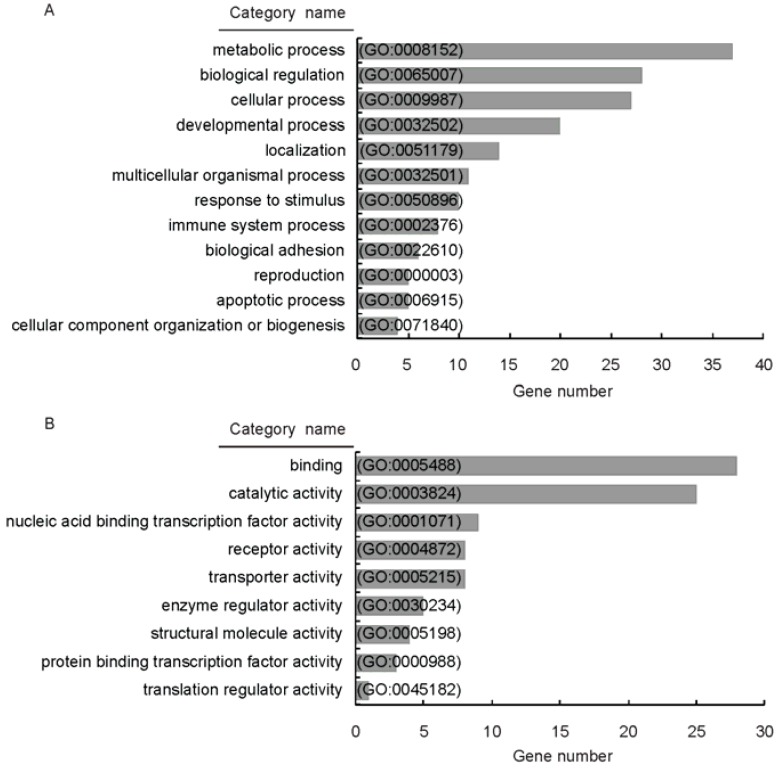
GO terms analysis of miR-196a biological function based on the predicted targets. (**A**) GO biological processes that were significantly overrepresented were generated from the annotations of biological processes; (**B**) GO molecular functions that were significantly overrepresented were generated from the annotations of molecular function. The target genes of miR-196a were predicted with TargetScan and PicTar, 74 overlapped targets were used for GO term enrichment analysis.

To further explore the potential biological roles of miR-196a, the TargetScan and PicTar algorithms were used to predict miR-196a targets. A total of 295 targets were predicted with TargetScan and 162 targets were predicted with PicTar; among these targets, 74 overlapped genes were used for GO term enrichment analysis and KEGG pathway analysis. The results showed that the overlapped targets were enriched in 12 categories including metabolic process, biological regulation, cellular process, developmental process, localization, response to stimulus, multicellular organismal process, immune system process, biological adhesion, reproduction, cellular component organization or biogenesis and apoptotic process (Figure 2A). These categories of biological processes refer to a number of specific molecular functions including binding, catalytic activity, receptor activity, nucleic acid binding transcription factor activity, transporter activity, structural molecule activity, enzyme regulator activity, protein binding transcription factor activity and translation regulator activity (Figure 2B). In addition, the overlapped targets of miR-196a were enriched in several pathways including glioma, focal adhesion, ECM–receptor interaction, gap junction, prostate cancer, GnRH signaling pathway, melanogenesis, and insulin signaling pathway (Table 3). These data indicate that miR-196a exhibits various biological functions.

**Table 3:** The KEGG pathways that are enriched with predicted targets of miR-196a by DAVID.

Pathways	Related Genes	*p*-Value
Glioma	calmodulin 3 (phosphorylase kinase, delta); calmodulin 2 (phosphorylase kinase, delta); calmodulin 1 (phosphorylase kinase, delta); neuroblastoma RAS viral (v-ras) oncogene homolog; platelet-derived growth factor receptor, alpha polypeptide	0.0182
Focal adhesion	collagen, type I, alpha 1; collagen, type I, alpha 2; collagen, type III, alpha 1; platelet-derived growth factor receptor, alpha polypeptide	0.0275
ECM–receptor interaction	collagen, type I, alpha 1; collagen, type I, alpha 2; collagen, type III, alpha 1	0.0312
Gap junction	adenylatecyclase 9; neuroblastoma RAS viral (v-ras) oncogene homolog; platelet-derived growth factor receptor, alpha polypeptide	0.0347
Prostate cancer	cyclin-dependent kinase inhibitor 1B (p27, Kip1); neuroblastoma RAS viral (v-ras) oncogene homolog; platelet-derived growth factor receptor, alpha polypeptide	0.0347
GnRH signaling pathway	adenylatecyclase 9; calmodulin 3 (phosphorylase kinase, delta); calmodulin 2 (phosphorylase kinase, delta); calmodulin 1 (phosphorylase kinase, delta); neuroblastoma RAS viral (v-ras) oncogene homolog	0.0414
Melanogenesis	adenylatecyclase 9; calmodulin 3 (phosphorylase kinase, delta); calmodulin 2 (phosphorylase kinase, delta); calmodulin 1 (phosphorylase kinase, delta); neuroblastoma RAS viral (v-ras) oncogene homolog	0.0422

KEGG pathway analysis was based on the predicted targets of miR-196a. The target genes of miR-196a were predicted with TargetScan and PicTar, 74 overlapped targets were used for KEGG pathway analysis with DAVID.

### 3.3. Generation and Identification of Recombinant Adenovirus Ad-miR-196a

**Figure 3:**
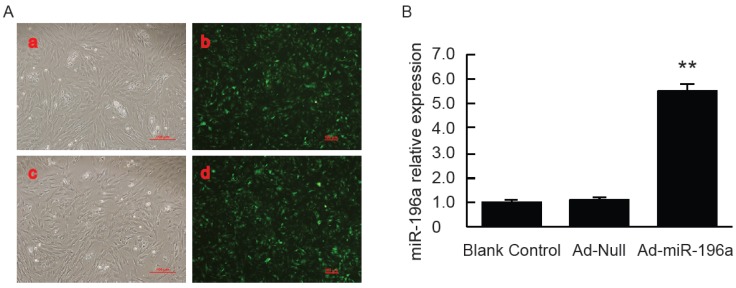
Overexpression of miR-196a in porcine primary adipocytes with Ad-miR-196a. (**A**) Porcine primary adipocytes infected with Ad-miR-196a or Ad-Null for 2 days were observed under a fluorescence microscope: a and b, adipocytes infected with Ad-Null (a × 200, b × 100); c and d, adipocytes infected with Ad-miR-196a (c × 200, d × 100); (**B**) miR-196a expression was detected by qPCR and normalized to U6 (*n* = 6 independent experiments). * = *p* < 0.05; ** = *p* < 0.01.

To study the potential roles of miR-196a in swine, recombinant adenovirus vector expressing miR-196a (Ad-miR-196a) was prepared. Porcine primary preadipocytes were then infected with Ad-miR-196a or Ad-Null for two days. Afterwards, the infected cells were observed under fluorescence microscope, and the expression of miR-196a was examined with qPCR. The infection efficiency was considered as the percentage of GFP expressed cell number to total cell number per view. As shown in Figure 3A, most of the cells in the view were GFP-positive, which led us to believe the infection efficiency was above 90%. More importantly, miR-196a expression was significantly elevated in cells infected with Ad-miR-196a compared with control cells (Figure 3B), representing an effective overexpression of miR-196a with Ad-miR-196a in porcine primary preadipocytes.

### 3.4. Roles of miR-196a in Porcine Adipocyte Development

**Figure 4:**
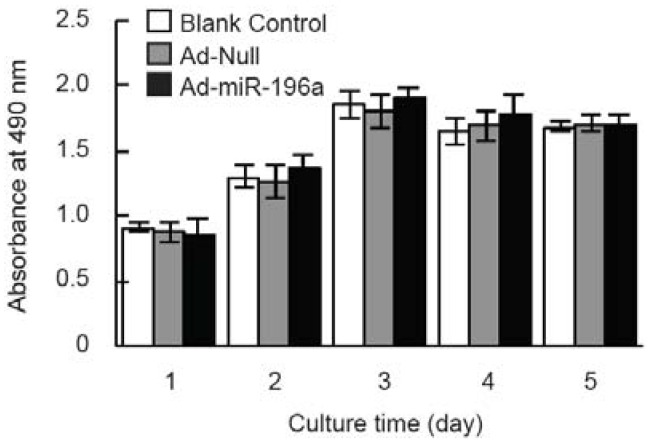
Proliferation of porcine preadipocytes accessed by MTT assay. Porcine preadipocytes infected with Ad-miR-196a or Ad-Null were cultured for 5 days, cell proliferation was assessed with MTT assay, normal cells as blank control (*n* = 12 in each group), *p* < 0.05 was considered as statistically significant.

**Figure 5:**
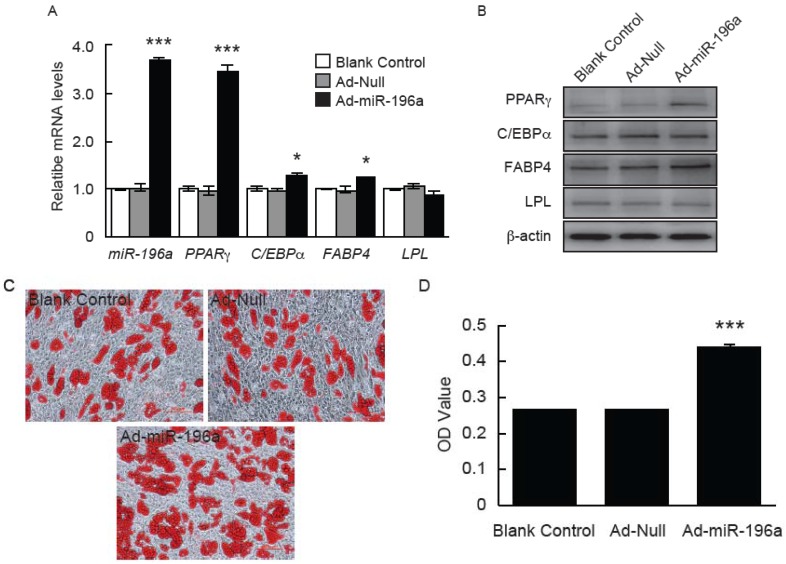
Overexpression of miR-196a promotes porcine adipogenesis. (**A**) Increased adipogenesis genes in preadipocytes infected with Ad-miR-196a. After adipogenic induction, the expression of adipocytes specific genes including PPARγ, C/EBPα, FABP4 and LPL was detected with qPCR at day 5 (*n* = 6 independent experiments). * = *p* < 0.05; ** = *p* < 0.01; *** = *p* < 0.001; (**B**) Protein levels of PPARγ, C/EBPα, FABP4 and LPL genes examined with Western blotting; (**C**) The morphological changes and lipid accumulation of porcine differentiated adipocytes observed by Oil Red O staining (a–c, ×200): a, Blank control; b, Ad-Null control; c, Ad-miR-196a group; (**D**) Cellular lipid content detected by Oil Red O extraction method. The cellular lipid contents were determined as 500 nm absorbance of Oil Red O (*n* = 6 independent experiments). * = *p* < 0.05; ** = *p* < 0.01; *** = *p* < 0.001.

Based on the robust increase of miR-196a expression in adipose tissue of adult pigs and the predicted roles of miR-196a on cell development, we proposed that miR-196a may play a role in the regulation of adipocyte development. To test this hypothesis, the proliferation and differentiation of porcine primary adipocytes were detected after overexpressing miR-196a. As shown in Figure 4, after five days’ infection with Ad-miR-196a in porcine preadipocytes, no significant proliferation changes were observed suggesting overexpression of miR-196a had no effect on porcine preadipocyte proliferation. However, the mRNA levels of adipocytes’ specific genes including PPARγ, C/EBPα and FABP4 were remarkably increased in miR-196a overexpressing cells (Figure 5A). Consistent with this, an obvious increase of protein levels of PPARγ and FABP4 were also detected in Ad-miR-196a infected cells by Western blotting, while the protein levels of C/EBPα and LPL genes showed no obvious change (Figure 5B). Meanwhile, Oil red O staining and extraction method was used to observe morphological changes and detect triglyceride content at day five of differentiation. Increased accumulation of lipid droplets and triglyceride levels were observed in Ad-miR-196a infected cells compared with control groups (Figure 5C,D), indicating overexpression of miR-196a stimulates porcine preadipocyte differentiation. These data demonstrated that miR-196a promotes porcine preadipocyte differentiation without affecting proliferation.

## 4. Discussion

Adipose tissue is not only an important energy storage organ, but also a key endocrine organ involved in maintaining a stable internal environment and the secretion of hormones and cytokines [38]. As the main component of adipose tissue, dysregulation of adipocyte proliferation or differentiation may cause obesity and other high risk metabolic diseases such as type 2 diabetes and hyperlipidemia [39]. Adipocyte proliferation and differentiation processes are influenced by various cytokines and hormones [40,41]. 

As post-transcriptional regulators, miRNAs not only influence cell development but also play important roles in various diseases [42,43,44]. The earliest research of miRNA in adipogenesis was performed in human primary preadipocytes, and miR-143 was reported to improve human adipogenesis by targeting ERK5 (Extracellular-signal-regulated kinase 5) [12]. As such, more and more miRNAs were found to play important roles in regulation of adipocyte development and its functions. 

In 3T3-L1 cells, overexpression of miR-17-92 cluster [10] and miR-103 [11] accelerates adipogenesis. Whereas, overexpression of let-7 [9], miR-27b [8] and miR-448 [7] inhibits 3T3-L1 adipogenesis by targeting HMGA2 (high-mobility group AT-hook 2), PPARγ and klf5 (kruppel-like factor 5), respectively. In human preadipocytes, overexpression of miR-130 suppresses adipogenesis by inhibiting PPARγ expression [13]. Moreover, miR-143 [12] and miR-519d [14] promote adipogenesis by targeting ERK5 and PPARα, respectively. Furthermore, miR-193b-365 cluster was demonstrated to be a key regulator of brown fat development, and blocking of the miR-193a-365 cluster in primary mouse preadipocytes markedly impaired brown adipocyte differentiation by enhancing Runx1t1 expression [45]. These reports demonstrate that miRNAs exhibit differential expression among different species and play important roles in the regulation of adipogenesis. However, little is known about miRNAs’ functions in porcine adipogenesis.

So far, the studies of miRNA in adipose biology have been mostly performed with human and mouse cells, and porcine primary adipocytes are seldom used. However, porcine adipocytes are easy to access and can easily induce differentiation. In addition, pigs are more close to humans in genetics and appear to have a higher fat deposition capability compared with other species [46,47,48]. Therefore, porcine primary adipocytes are the optimal cell model for adipocyte biology research. 

In this report, for the first time, we demonstrated that miR-196a was widely and differentially expressed in swine tissues including heart, liver, lung, kidney, spleen, skeletal muscle and adipose tissue (Figure 1). In piglets, miR-196a was predominantly expressed in kidney and skeletal muscle tissues. In contrast, miR-196a was mainly expressed in adipose tissue and liver of adult pigs. Our results were consistent with previous studies in which miR-196a was enriched in the kidney and reproductive tissues of bovine and mouse [16,49]. The differential expression levels of miR-196a in tissues from piglets and adult pigs show miR-196a may play a role in porcine tissue development. Especially, the highest levels of miR-196a were found in adult pig adipose tissue and liver, the main locations of fat metabolism, leading us to hypothesize that miR-196a has functions in adipose development.

In accordance with the previous report that miR-196a was upregulated in mouse SVF cells during adipogenic differentiation [50], increased levels of miR-196a were observed in adipose tissue from adult pigs compared with piglets (Table 1). Further bioinformatics function analysis revealed that potential targets of miR-196a were significantly enriched in skeletal system development, organ morphogenesis, the cellular macromolecule metabolic process and the macromolecule metabolic process. The potential function in skeletal system development was coordinated with the high expression of miR-196a in skeletal muscle tissue of piglets. Although some of miR-196a’s potential functions have been confirmed in previous reports, particularly in respect to cell proliferation and differentiation, tumorigenesis, tail regeneration, immunology and virus defense [15,16,17,18,19,20,21,22], none of these potential biological roles have been well studied in pigs. 

To investigate whether miR-196a plays a role in regulation of adipocyte development, porcine preadipocytes overexpressing miR-196a were used for cell proliferation and differentiation assay. Our data demonstrated that miR-196a overexpression enhanced adipocyte differentiation (Figure 5), without influencing cell proliferation (Figure 4). Consistently, ASO (antisense oligonucleotide) against miR-196a suppressed the expression of PPARγ in mouse SVF (stromal vascular fraction) cells followed by adipogenic induction [50]. However, our results are in disagreement with previous findings [22], which demonstrated that overexpression of miR-196a decreased hASC (human adipose tissue-derived mesenchymal stem cells) proliferation and enhanced osteogenic differentiation, without affecting adipogenic differentiation. It may be due to the differences among species or cell types. Moreover, it is known that the same miRNAs may play different roles in different development stages of the same cells, or in the same development stages of different cells. 

However, the mechanisms by which miR-196a affect adipogenesis in swine are still not clear. The miR-196a-mediated upregulation of adipogenic markers suggests it might function through indirect pathways because miRNA normally suppresses its targets. The previous studies found miR-196a is involved in regulation of BMP [17], WNT [21] and AKT [20] pathways. These three pathways and the predicted targets of miR-196a enriched pathways could be the potential targets of miR-196a function. Further research is needed to better understand the mechanisms of miR-196a mediated adipogenesis in swine.

## 5. Conclusions

Our data demonstrated that miR-196a was widely and differentially expressed in pig tissues for the first time, and adenovirus mediated overexpression of miR-196a in porcine adipocytes showed miR-196a enhanced porcine adipocyte differentiation, both in terms of morphology and genetic levels; while no proliferation changes were found in this process. The present study provides new insights into the biological roles of miR-196a, and detailed functional analyses need to be conducted to understand the molecular aspects of miR-196a in porcine adipogenesis in the future.
